# Human brown adipose tissue is not enough to combat cardiometabolic diseases

**DOI:** 10.1172/JCI175288

**Published:** 2023-12-01

**Authors:** André C. Carpentier, Denis P. Blondin

**Affiliations:** 1 Division of Endocrinology and; 2 Neurology, Department of Medicine, Centre de recherche du Centre hospitalier universitaire de Sherbrooke, Université de Sherbrooke, Sherbrooke, Quebec, Canada.

Brown adipose tissue (BAT) stands out as the sole tissue able to substantially expend energy by leveraging uncoupling protein 1 (UCP1) to increase proton permeability through the inner mitochondrial membrane. The renewed interest in BAT in adult humans resulted from the growing use of ^18^F-fluoro-deoxyglucose (^18^FDG) PET coupled with either CT or MRI, and BAT continues to be defined and quantified in vivo according to features visualized through these imaging techniques (1). Inverse associations between the presence of ^18^FDG-positive adipose tissue and obesity, visceral fat, hyperglycemia, and diagnosis of type 2 diabetes (T2D) have been widely reported (2) and have led to the hypothesis that BAT metabolic dysfunction is a plausible contributor to obesity, T2D, and cardiometabolic diseases. Rodent studies have provided strong support for this hypothesis, as cold- and/or β-adrenergic agonist-stimulated BAT was shown to be a major driver of systemic clearance of glucose and circulating fat. Although BAT is unquestionably considered one of the hottest current targets for the treatment of obesity, T2D, and cardiometabolic complications, we posit that, in contrast to rodents, human BAT is not sufficient to combat cardiometabolic diseases.

## BAT glucose uptake does not reflect BAT thermogenesis

The first problem with the suggestion that BAT may play a role in cardiometabolic diseases involves BAT glucose uptake, which is used to define and quantify BAT’s presence in almost all human studies. However, glucose uptake does not necessarily indicate BAT thermogenic activity. Most of the glucose taken up by BAT is metabolized to lactate and intracellular triglycerides (TG) (1, 3) and very little is used directly as a substrate for thermogenesis. Furthermore, insulin stimulates BAT glucose uptake without eliciting changes in tissue blood flow (4), suggesting that the physiological regulation of BAT glucose uptake and metabolism may act, in part, independent of thermogenesis. Cold-induced BAT glucose uptake is profoundly reduced in older, overweight men with or without T2D compared to lean healthy men, but without reduction in BAT thermogenesis as assessed by ^11^C-acetate PET (5). Further, a fructose-overfeeding regimen, capable of inducing visceral obesity, postprandial dyslipidemia, and systemic insulin resistance when prolonged for several weeks (6), selectively reduced cold-induced BAT glucose uptake without eliciting a change in BAT thermogenesis, measured by ^11^C-acetate PET after only two weeks of overfeeding (7). This reduction in BAT glucose uptake also occurred without any changes in circulating insulin, glucose, lipids, or change in the gut microbiota. Fasting-induced insulin resistance has also been associated with reduced BAT glucose uptake (8). Thus, reduced BAT glucose uptake is not necessarily indicative of reduced BAT thermogenesis, but may be a very early and sensitive indicator of nutritional and environmental exposures leading to dysmetabolism.

## Adults have small volumes of BAT

The second problem with BAT as a major contributor to cardiometabolic disorders relates to its very low volume in humans compared with rodents. In humans, ^18^FDG-positive BAT volume typically ranges between 50 to 150 mL in most individuals acutely exposed to cold (9), and can increase an additional approximately 40% when rigorous cold acclimation is applied for 10 days to 4 weeks (10). Radiological mapping of adipose tissue depots containing ^18^FDG-positive BAT suggests upper ranges of 550 to 2550 mL, although only a small fraction of these volumes is demonstrably active metabolically (11). A hypothetical scenario in which individuals are exposed to a mild cold stimulus and have three different volumes of metabolically active BAT, 150 mL, 550 mL, or 2,550 mL, results in energy consumption rates of 12 kcal/24 hours, 46 kcal/24 hours, and 211 kcal/24 hours respectively, assuming sustained energy consumption and using BAT oxygen consumption rates derived from mild cold-exposure experiments involving ^15^O-O_2_ PET (12). If calculated as a percentage of the increase in whole body energy expenditure stimulated from mild cold-exposure, which is approximately 1,440 kcal/24 hours, the values correspond to 0.8%, 3%, and 15% (5). Thus, for most individuals, current estimates of BAT thermogenic capacity are not compatible with a notable contribution of BAT to energy balance, even assuming continuous mild cold exposure, as performed in experimental settings. Likewise, BAT in humans contributes less than 1% to the clearance of systemic glucose, nonesterified fatty acids (NEFA), and plasma TG or dietary fatty acids from chylomicron TG, even under a nonrealistic degree of sustained cold exposure (1).

## Cold-induced thermogenesis occurs from a coordinated multi-organ response

**Figure 1:**
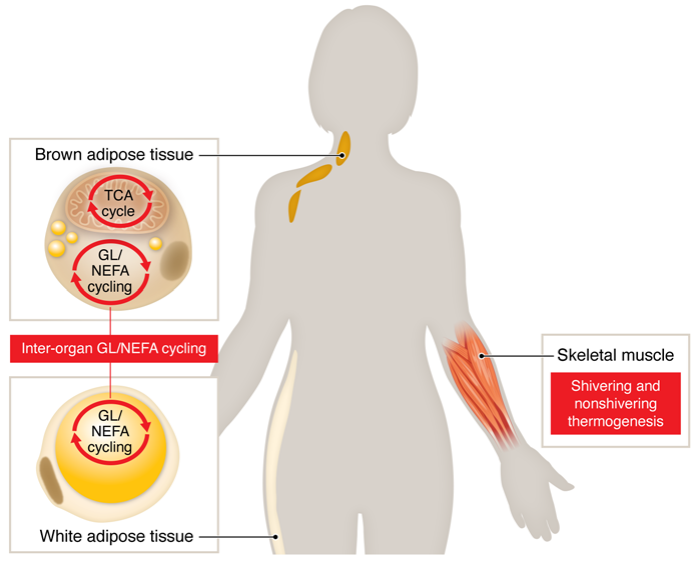
Cold-induced thermogenesis is a multi-organ process. BAT thermogenesis from uncoupling protein 1–mediated mitochondrial respiration likely contributes only a small fraction (*i.e.*, less than 3%) of whole-body, cold-induced thermogenesis in humans. Skeletal muscle shivering and nonshivering thermogenesis drive most of the whole-body thermogenic response to cold. Glycerolipid and nonesterified fatty acid (GL/NEFA) substrate cycling activated in both WAT and BAT leads to systemic NEFA mobilization, likely driving NEFA reesterification in other organs (termed inter-organ GL/NEFA cycling) and also contributing to systemic energy expenditure.

A multi-organ response drives the large increase in whole body energy expenditure during experimental cold-exposure conditions, which is same in young, healthy, and lean individuals and in older, overweight individuals, without or with T2D (5). Muscle glucose uptake and nonshivering and shivering metabolic activity is largely unavoidable in humans exposed to mild cold, and is mostly responsible for the metabolic benefits of cold exposure (1, 13). One other potential source of energy expenditure is reesterification of the increased systemic NEFA flux from white adipose tissues (WAT) by other tissues (such as the liver) that occurs with cold exposure and β-adrenergic agonist treatment alike (14, 15). While browning of WAT has been suggested to contribute to energy expenditure after chronic cold exposure, the typical increase in thermogenesis evident in BAT is not detectable in WAT in vivo using ^11^C-acetate (16) or ex vivo using respirometry (17), in contrast to marked WAT browning seen in rodents. This discrepancy is not due to the lack of molecular sensitivity in PET imaging, which is in the femto, or 10^-15^, to picomolar, or 10^-12^, range, as it is by far the most sensitive method to measure metabolic fluxes in vivo, and is at least as sensitive as the most sensitive in vitro and ex vivo methods. UCP1-expressing cells, like other adipocytes in WAT, could, however, contribute to the glycerolipid-NEFA (GL/NEFA) substrate cycling by providing larges fluxes of NEFA. These fluxes of NEFA could be, in part, reesterified in other adipocytes or other organs (17). BAT also likely contributes to this inter-organ GL/NEFA substrate cycling as it rapidly mobilizes up to several grams of its own TG content upon acute cold exposure (1). Recycling of this large amount of NEFA in situ and/or in other organs may contribute to energy dissipation. In support of the contribution of this GL/NEFA cycling to cold-induced thermogenesis, interrupting BAT and WAT intracellular lipolysis with nicotinic acid during cold exposure, thereby reducing this cycling, leads to increased muscle shivering while total body energy expenditure remains the same (18). Shivering and BAT- or WAT-driven GL/NEFA cycling-mediated thermogenesis thus appear to coordinate cold-induced thermogenic response (Figure 1).

## Human thermogenesis is unlikely a BAT-dependent endocrine function

BAT-mediated mechanisms other than direct thermogenesis and energy substrate metabolism have been proposed. These include the secretion of biologically active metabolites or proteins that could act as endocrine factors to drive the metabolic benefits observed with BAT activation during cold or β-adrenergic stimulation (19). There is a priori no biological feature of BAT other than its unique capacity to produce heat through UCP1-dependent proton leak that suggests it as a target for the treatment of metabolic diseases. Indeed, none of these inter-tissue mediators, termed ‘batokines’, are specifically secreted by BAT or are directly demonstrated to be responsible for cold-induced metabolic improvements. The relative contribution of BAT versus WAT, skeletal muscles, liver, and/or immune cells to systemic levels of these ‘batokines’ is currently unknown in humans, but is likely to be small. Thus, while not formally excluded at this time, a role for BAT as an endocrine organ is not the most elegant explanation for the metabolic benefits of cold exposure.

## BAT thermogenic characteristics are insufficient to treat obesity

BAT is an exciting tissue by virtue of its unique capacity to dissipate energy to produce heat as its principal metabolic product. However, our knowledge of human BAT pathophysiology is overtly reliant on BAT glucose uptake that is often uncoupled from BAT thermogenesis. The latter is almost never directly measured in vivo in human or even in animal investigations. Correlation is not causation, and the association between low ^18^FDG-positive BAT volumes and cardiometabolic diseases is likely driven by subtle changes in insulin sensitivity and/or unmeasured dysmetabolic environmental exposures. Cold acclimation and/or β-adrenergic agonists can clearly activate and recruit BAT while simultaneously increasing energy expenditure and substrate utilization (10, 14, 15, 20). However, the small volume of BAT makes it unlikely to drive, by itself, the systemic metabolic benefits of cold exposure in humans. BAT never acts alone, as skeletal muscles, WAT, and probably other organs, contribute markedly to the changes in human metabolism observed under conditions where BAT is also metabolically activated (1). Coordinated activation of GL/NEFA substrate cycling, and likely other metabolic cycles from WAT, BAT, and other organs occurs and appears to play an important role in cold-induced thermogenesis. Indeed, in the absence of skeletal muscle shivering activity, there is no cold-induced improvement in peripheral insulin sensitivity (13). BAT thermogenic activity is the tip of a much larger multi-organ cold- and β-adrenergic agonist–stimulated response that needs to be better understood in humans before being exploited as a target for the treatment of cardiometabolic diseases.

## Version 1 12/01/2023

Electronic publication
